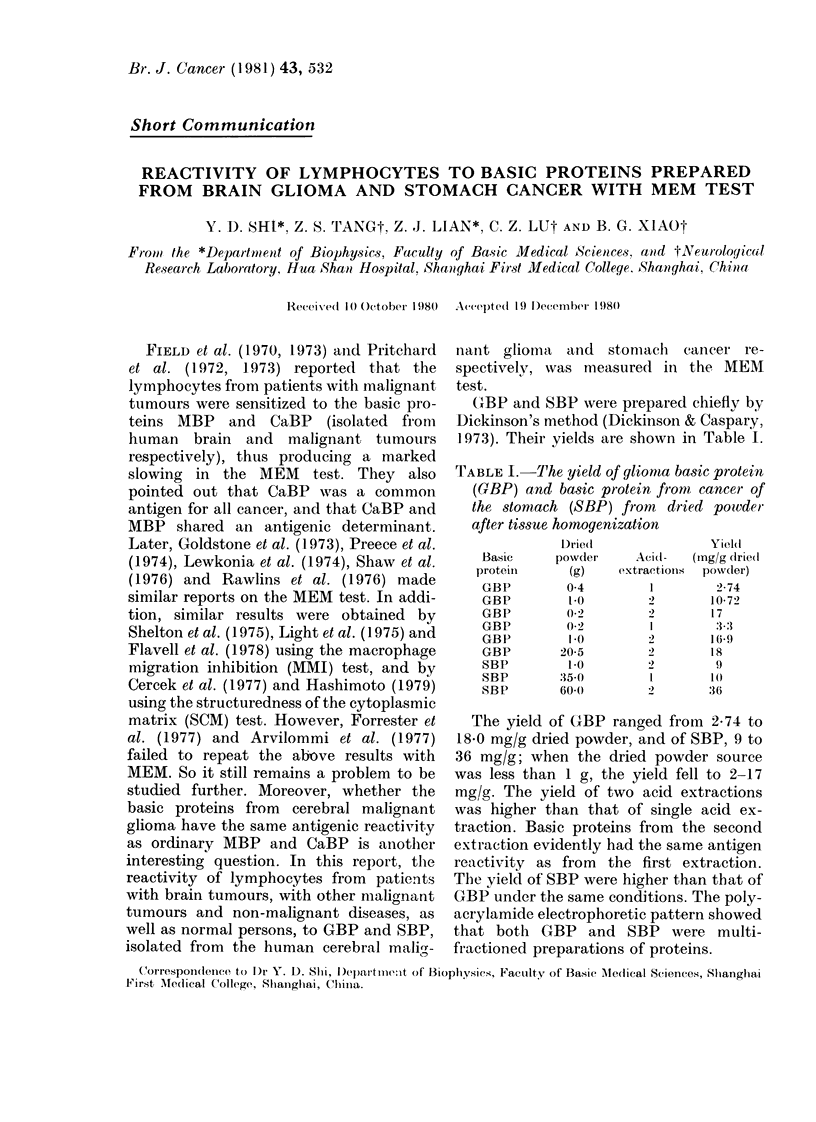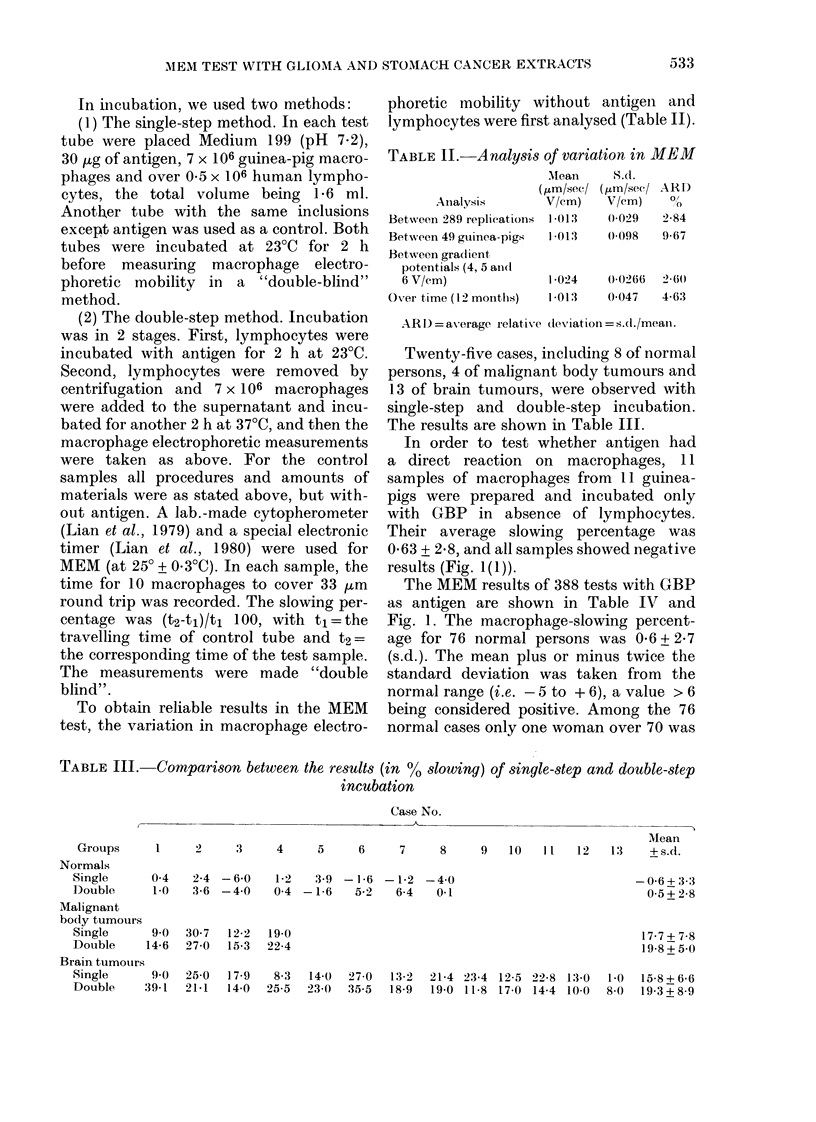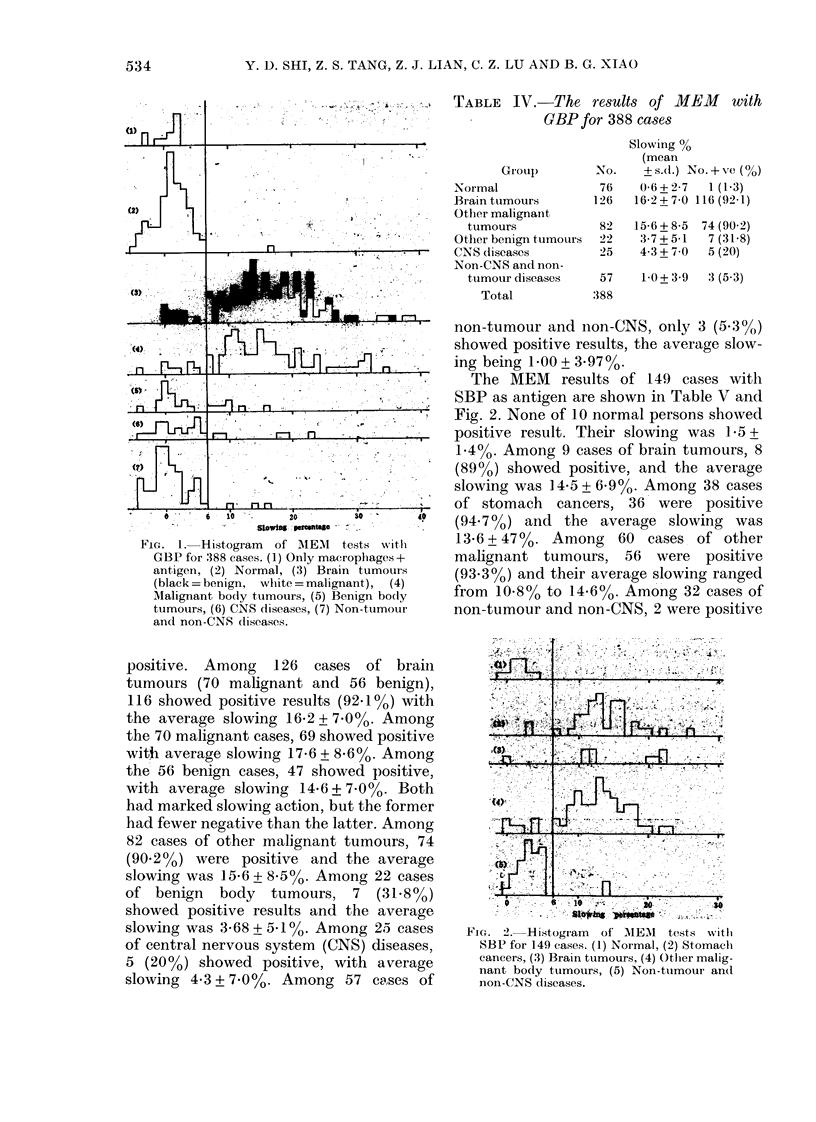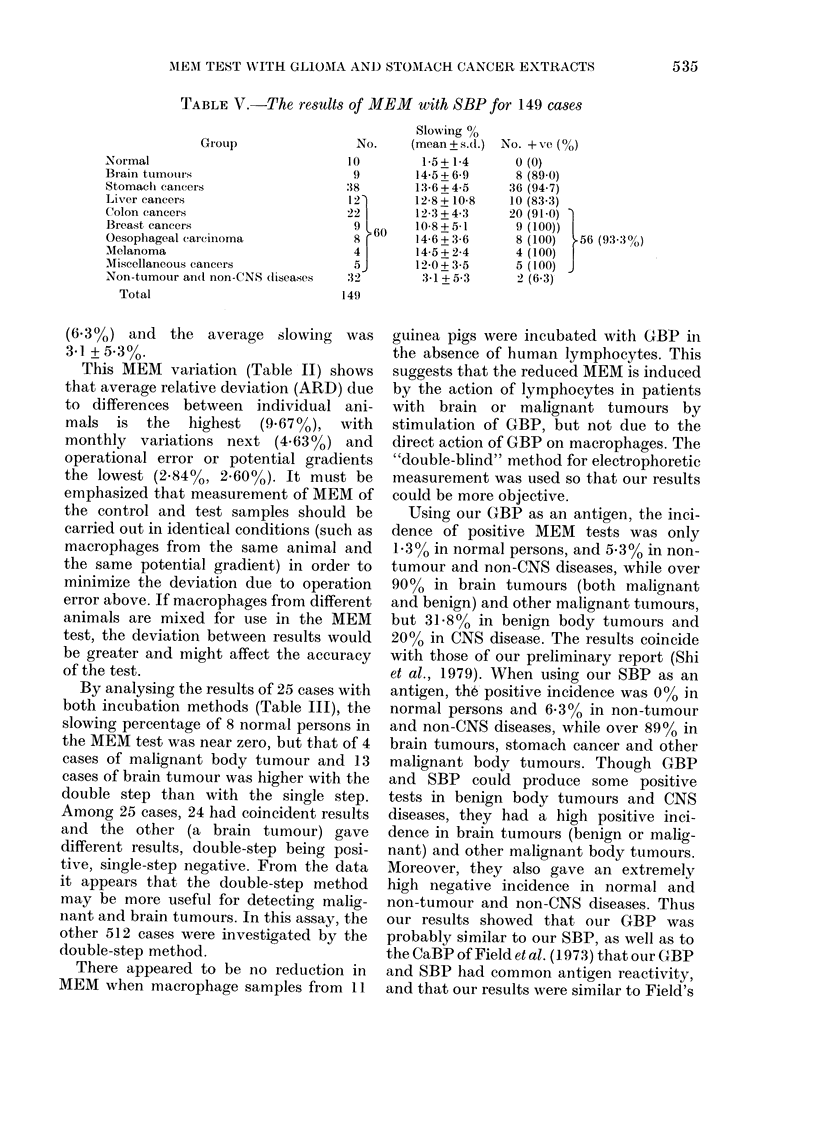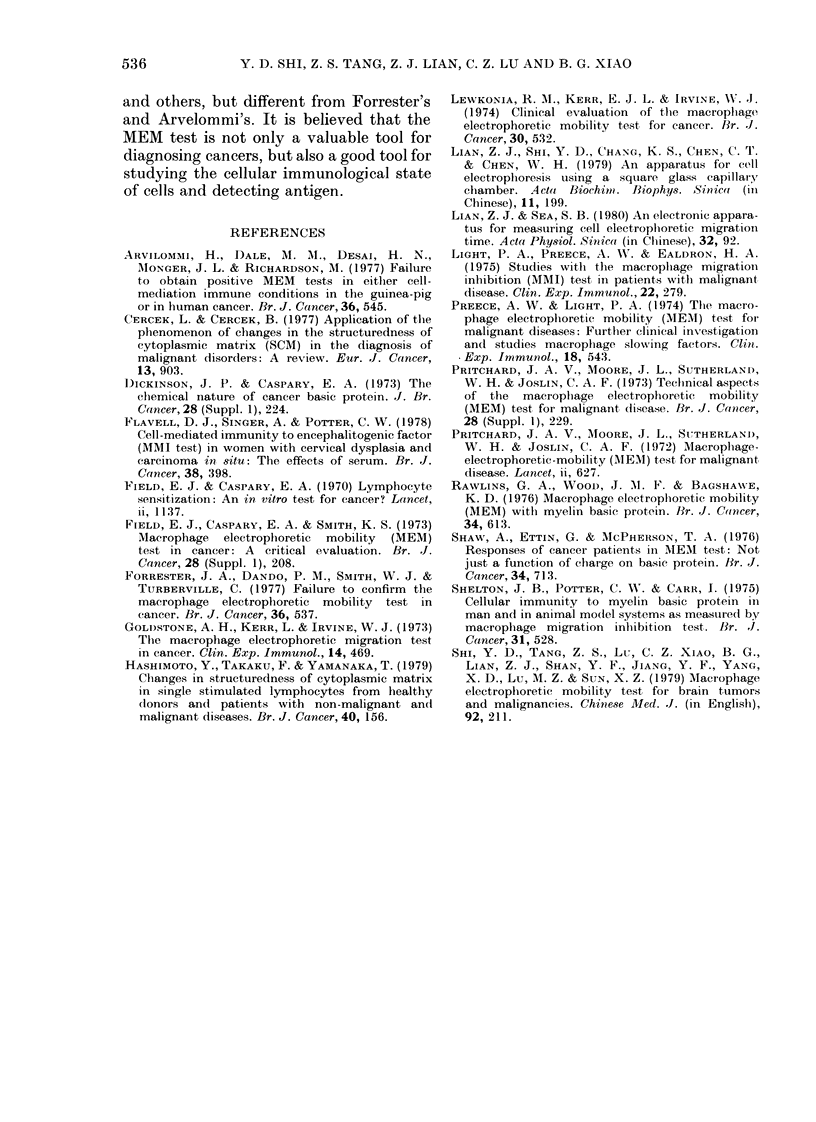# Reactivity of lymphocytes to basic proteins prepared from brain glioma and stomach cancer with MEM test.

**DOI:** 10.1038/bjc.1981.76

**Published:** 1981-04

**Authors:** Y. D. Shi, Z. S. Tang, Z. J. Lian, C. Z. Lu, B. G. Xiao


Br. J. Cancer (1981) 43, 532

*Short Communication*

### REACTIVITY OF LYMPHOCYTES TO BASIC PROTEINS PREPARED FROM BRAIN GLIOMA AND STOMACH CANCER WITH MEM TEST

Y. D. SHI*, Z. S. TANG†, Z. J. LIAN*, C. Z. LU† AND B. G. XIAO†

*From the *Department of Biophysics, Faculty of Basic Medical Sciences, and †Neurological Research Laboratory, Hua Shan Hospital, Shanghai First Medical College, Shanghai, China*

Received 10 October 1980 Accepted 19 December 1980

FIELD *et al.* (1970, 1973) and Pritchard *et al.* (1972, 1973) reported that the lymphocytes from patients with malignant tumours were sensitized to the basic proteins MBP and CaBP (isolated from human brain and malignant tumours respectively), thus producing a marked slowing in the MEM test. They also pointed out that CaBP was a common antigen for all cancer, and that CaBP and MBP shared an antigenic determinant. Later, Goldstone *et al.* (1973), Preece *et al.* (1974), Lewkonia *et al.* (1974), Shaw *et al.* (1976) and Rawlins *et al.* (1976) made similar reports on the MEM test. In addition, similar results were obtained by Shelton *et al.* (1975), Light *et al.* (1975) and Flavell *et al.* (1978) using the macrophage migration inhibition (MMI) test, and by Cercek *et al.* (1977) and Hashimoto (1979) using the structuredness of the cytoplasmic matrix (SCM) test. However, Forrester *et al.* (1977) and Arvilommi *et al.* (1977) failed to repeat the above results with MEM. So it still remains a problem to be studied further. Moreover, whether the basic proteins from cerebral malignant glioma have the same antigenic reactivity as ordinary MBP and CaBP is another interesting question. In this report, the reactivity of lymphocytes from patients with brain tumours, with other malignant tumours and non-malignant diseases, as well as normal persons, to GBP and SBP, isolated from the human cerebral malignant glioma and stomach cancer respectively, was measured in the MEM test.

GBP and SBP were prepared chiefly by Dickinson's method (Dickinson & Caspary, 1973). Their yields are shown in Table I.

TABLE I.—*The yield of glioma basic protein (GBP) and basic protein from cancer of the stomach (SBP) from dried powder after tissue homogenization*

| Basic protein | Dried powder (g) | Acid-extractions | Yield (mg/g dried powder) |
|---|---|---|---|
| GBP | 0·4 | 1 | 2·74 |
| GBP | 1·0 | 2 | 10·72 |
| GBP | 0·2 | 2 | 17 |
| GBP | 0·2 | 1 | 3·3 |
| GBP | 1·0 | 2 | 16·9 |
| GBP | 20·5 | 2 | 18 |
| SBP | 1·0 | 2 | 9 |
| SBP | 35·0 | 1 | 10 |
| SBP | 60·0 | 2 | 36 |

The yield of GBP ranged from 2·74 to 18·0 mg/g dried powder, and of SBP, 9 to 36 mg/g; when the dried powder source was less than 1 g, the yield fell to 2–17 mg/g. The yield of two acid extractions was higher than that of single acid extraction. Basic proteins from the second extraction evidently had the same antigen reactivity as from the first extraction. The yield of SBP were higher than that of GBP under the same conditions. The polyacrylamide electrophoretic pattern showed that both GBP and SBP were multifractioned preparations of proteins.

Correspondence to Dr Y. D. Shi, Department of Biophysics, Faculty of Basic Medical Sciences, Shanghai First Medical College, Shanghai, China.

In incubation, we used two methods:

(1) The single-step method. In each test tube were placed Medium 199 (pH 7·2), 30 $\mu$g of antigen, $7 \times 10^6$ guinea-pig macrophages and over $0{\cdot}5 \times 10^6$ human lymphocytes, the total volume being 1·6 ml. Another tube with the same inclusions except antigen was used as a control. Both tubes were incubated at 23°C for 2 h before measuring macrophage electrophoretic mobility in a "double-blind" method.

(2) The double-step method. Incubation was in 2 stages. First, lymphocytes were incubated with antigen for 2 h at 23°C. Second, lymphocytes were removed by centrifugation and $7 \times 10^6$ macrophages were added to the supernatant and incubated for another 2 h at 37°C, and then the macrophage electrophoretic measurements were taken as above. For the control samples all procedures and amounts of materials were as stated above, but without antigen. A lab.-made cytopherometer (Lian *et al.*, 1979) and a special electronic timer (Lian *et al.*, 1980) were used for MEM (at 25° ± 0·3°C). In each sample, the time for 10 macrophages to cover 33 $\mu$m round trip was recorded. The slowing percentage was $(t_2\text{-}t_1)/t_1$ 100, with $t_1$ = the travelling time of control tube and $t_2$ = the corresponding time of the test sample. The measurements were made "double blind".

To obtain reliable results in the MEM test, the variation in macrophage electrophoretic mobility without antigen and lymphocytes were first analysed (Table II).

TABLE II.—*Analysis of variation in MEM*

| Analysis | Mean ($\mu$m/sec/ V/cm) | S.d. ($\mu$m/sec/ V/cm) | ARD % |
|---|---|---|---|
| Between 289 replications | 1·013 | 0·029 | 2·84 |
| Between 49 guinea-pigs | 1·013 | 0·098 | 9·67 |
| Between gradient potentials (4, 5 and 6 V/cm) | 1·024 | 0·0266 | 2·60 |
| Over time (12 months) | 1·013 | 0·047 | 4·63 |

ARD = average relative deviation = s.d./mean.

Twenty-five cases, including 8 of normal persons, 4 of malignant body tumours and 13 of brain tumours, were observed with single-step and double-step incubation. The results are shown in Table III.

In order to test whether antigen had a direct reaction on macrophages, 11 samples of macrophages from 11 guinea-pigs were prepared and incubated only with GBP in absence of lymphocytes. Their average slowing percentage was 0·63 ± 2·8, and all samples showed negative results (Fig. 1(1)).

The MEM results of 388 tests with GBP as antigen are shown in Table IV and Fig. 1. The macrophage-slowing percentage for 76 normal persons was 0·6 ± 2·7 (s.d.). The mean plus or minus twice the standard deviation was taken from the normal range (*i.e.* −5 to +6), a value > 6 being considered positive. Among the 76 normal cases only one woman over 70 was

TABLE III.—*Comparison between the results (in % slowing) of single-step and double-step incubation*

| Groups | Case No. 1 | 2 | 3 | 4 | 5 | 6 | 7 | 8 | 9 | 10 | 11 | 12 | 13 | Mean ± s.d. |
|---|---|---|---|---|---|---|---|---|---|---|---|---|---|---|
| Normals | | | | | | | | | | | | | | |
| Single | 0·4 | 2·4 | −6·0 | 1·2 | 3·9 | −1·6 | −1·2 | −4·0 | | | | | | −0·6 ± 3·3 |
| Double | 1·0 | 3·6 | −4·0 | 0·4 | −1·6 | 5·2 | 6·4 | 0·1 | | | | | | 0·5 ± 2·8 |
| Malignant body tumours | | | | | | | | | | | | | | |
| Single | 9·0 | 30·7 | 12·2 | 19·0 | | | | | | | | | | 17·7 ± 7·8 |
| Double | 14·6 | 27·0 | 15·3 | 22·4 | | | | | | | | | | 19·8 ± 5·0 |
| Brain tumours | | | | | | | | | | | | | | |
| Single | 9·0 | 25·0 | 17·9 | 8·3 | 14·0 | 27·0 | 13·2 | 21·4 | 23·4 | 12·5 | 22·8 | 13·0 | 1·0 | 15·8 ± 6·6 |
| Double | 39·1 | 21·1 | 14·0 | 25·5 | 23·0 | 35·5 | 18·9 | 19·0 | 11·8 | 17·0 | 14·4 | 10·0 | 8·0 | 19·3 ± 8·9 |

FIG. 1.—Histogram of MEM tests with GBP for 388 cases. (1) Only macrophages + antigen, (2) Normal, (3) Brain tumours (black = benign, white = malignant), (4) Malignant body tumours, (5) Benign body tumours, (6) CNS diseases, (7) Non-tumour and non-CNS diseases.

positive. Among 126 cases of brain tumours (70 malignant and 56 benign), 116 showed positive results (92·1%) with the average slowing 16·2 ± 7·0%. Among the 70 malignant cases, 69 showed positive with average slowing 17·6 ± 8·6%. Among the 56 benign cases, 47 showed positive, with average slowing 14·6 ± 7·0%. Both had marked slowing action, but the former had fewer negative than the latter. Among 82 cases of other malignant tumours, 74 (90·2%) were positive and the average slowing was 15·6 ± 8·5%. Among 22 cases of benign body tumours, 7 (31·8%) showed positive results and the average slowing was 3·68 ± 5·1%. Among 25 cases of central nervous system (CNS) diseases, 5 (20%) showed positive, with average slowing 4·3 ± 7·0%. Among 57 cases of

TABLE IV.—*The results of MEM with GBP for* 388 *cases*

| Group | No. | Slowing % (mean ± s.d.) | No. + ve (%) |
|---|---|---|---|
| Normal | 76 | 0·6 ± 2·7 | 1 (1·3) |
| Brain tumours | 126 | 16·2 ± 7·0 | 116 (92·1) |
| Other malignant tumours | 82 | 15·6 ± 8·5 | 74 (90·2) |
| Other benign tumours | 22 | 3·7 ± 5·1 | 7 (31·8) |
| CNS diseases | 25 | 4·3 ± 7·0 | 5 (20) |
| Non-CNS and non-tumour diseases | 57 | 1·0 ± 3·9 | 3 (5·3) |
| Total | 388 | | |

non-tumour and non-CNS, only 3 (5·3%) showed positive results, the average slowing being 1·00 ± 3·97%.

The MEM results of 149 cases with SBP as antigen are shown in Table V and Fig. 2. None of 10 normal persons showed positive result. Their slowing was 1·5 ± 1·4%. Among 9 cases of brain tumours, 8 (89%) showed positive, and the average slowing was 14·5 ± 6·9%. Among 38 cases of stomach cancers, 36 were positive (94·7%) and the average slowing was 13·6 ± 47%. Among 60 cases of other malignant tumours, 56 were positive (93·3%) and their average slowing ranged from 10·8% to 14·6%. Among 32 cases of non-tumour and non-CNS, 2 were positive

FIG. 2.—Histogram of MEM tests with SBP for 149 cases. (1) Normal, (2) Stomach cancers, (3) Brain tumours, (4) Other malignant body tumours, (5) Non-tumour and non-CNS diseases.

TABLE V.—*The results of MEM with SBP for* 149 *cases*

| Group | No. | | Slowing % (mean ± s.d.) | No. +ve (%) | |
|---|---|---|---|---|---|
| Normal | 10 | | 1·5 ± 1·4 | 0 (0) | |
| Brain tumours | 9 | | 14·5 ± 6·9 | 8 (89·0) | |
| Stomach cancers | 38 | | 13·6 ± 4·5 | 36 (94·7) | |
| Liver cancers | 12 | 60 | 12·8 ± 10·8 | 10 (83·3) | |
| Colon cancers | 22 | | 12·3 ± 4·3 | 20 (91·0) | 56 (93·3%) |
| Breast cancers | 9 | | 10·8 ± 5·1 | 9 (100)) | |
| Oesophageal carcinoma | 8 | | 14·6 ± 3·6 | 8 (100) | |
| Melanoma | 4 | | 14·5 ± 2·4 | 4 (100) | |
| Miscellaneous cancers | 5 | | 12·0 ± 3·5 | 5 (100) | |
| Non-tumour and non-CNS diseases | 32 | | 3·1 ± 5·3 | 2 (6·3) | |
| Total | 149 | | | | |

(6·3%) and the average slowing was 3·1 ± 5·3%.

This MEM variation (Table II) shows that average relative deviation (ARD) due to differences between individual animals is the highest (9·67%), with monthly variations next (4·63%) and operational error or potential gradients the lowest (2·84%, 2·60%). It must be emphasized that measurement of MEM of the control and test samples should be carried out in identical conditions (such as macrophages from the same animal and the same potential gradient) in order to minimize the deviation due to operation error above. If macrophages from different animals are mixed for use in the MEM test, the deviation between results would be greater and might affect the accuracy of the test.

By analysing the results of 25 cases with both incubation methods (Table III), the slowing percentage of 8 normal persons in the MEM test was near zero, but that of 4 cases of malignant body tumour and 13 cases of brain tumour was higher with the double step than with the single step. Among 25 cases, 24 had coincident results and the other (a brain tumour) gave different results, double-step being positive, single-step negative. From the data it appears that the double-step method may be more useful for detecting malignant and brain tumours. In this assay, the other 512 cases were investigated by the double-step method.

There appeared to be no reduction in MEM when macrophage samples from 11 guinea pigs were incubated with GBP in the absence of human lymphocytes. This suggests that the reduced MEM is induced by the action of lymphocytes in patients with brain or malignant tumours by stimulation of GBP, but not due to the direct action of GBP on macrophages. The "double-blind" method for electrophoretic measurement was used so that our results could be more objective.

Using our GBP as an antigen, the incidence of positive MEM tests was only 1·3% in normal persons, and 5·3% in non-tumour and non-CNS diseases, while over 90% in brain tumours (both malignant and benign) and other malignant tumours, but 31·8% in benign body tumours and 20% in CNS disease. The results coincide with those of our preliminary report (Shi *et al.*, 1979). When using our SBP as an antigen, the positive incidence was 0% in normal persons and 6·3% in non-tumour and non-CNS diseases, while over 89% in brain tumours, stomach cancer and other malignant body tumours. Though GBP and SBP could produce some positive tests in benign body tumours and CNS diseases, they had a high positive incidence in brain tumours (benign or malignant) and other malignant body tumours. Moreover, they also gave an extremely high negative incidence in normal and non-tumour and non-CNS diseases. Thus our results showed that our GBP was probably similar to our SBP, as well as to the CaBP of Field *et al.* (1973) that our GBP and SBP had common antigen reactivity, and that our results were similar to Field's and others, but different from Forrester's and Arvelommi's. It is believed that the MEM test is not only a valuable tool for diagnosing cancers, but also a good tool for studying the cellular immunological state of cells and detecting antigen.

### REFERENCES

Arvilommi, H., Dale, M. M., Desai, H. N., Monger, J. L. & Richardson, M. (1977) Failure to obtain positive MEM tests in either cell-mediation immune conditions in the guinea-pig or in human cancer. *Br. J. Cancer*, **36**, 545.

Cercek, L. & Cercek, B. (1977) Application of the phenomenon of changes in the structuredness of cytoplasmic matrix (SCM) in the diagnosis of malignant disorders: A review. *Eur. J. Cancer*, **13**, 903.

Dickinson, J. P. & Caspary, E. A. (1973) The chemical nature of cancer basic protein. *J. Br. Cancer*, **28** (Suppl. 1), 224.

Flavell, D. J., Singer, A. & Potter, C. W. (1978) Cell-mediated immunity to encephalitogenic factor (MMI test) in women with cervical dysplasia and carcinoma *in situ*: The effects of serum. *Br. J. Cancer*, **38**, 398.

Field, E. J. & Caspary, E. A. (1970) Lymphocyte sensitization: An *in vitro* test for cancer? *Lancet*, ii, 1137.

Field, E. J., Caspary, E. A. & Smith, K. S. (1973) Macrophage electrophoretic mobility (MEM) test in cancer: A critical evaluation. *Br. J. Cancer*, **28** (Suppl. 1), 208.

Forrester, J. A., Dando, P. M., Smith, W. J. & Turberville, C. (1977) Failure to confirm the macrophage electrophoretic mobility test in cancer. *Br. J. Cancer*, **36**, 537.

Goldstone, A. H., Kerr, L. & Irvine, W. J. (1973) The macrophage electrophoretic migration test in cancer. *Clin. Exp. Immunol.*, **14**, 469.

Hashimoto, Y., Takaku, F. & Yamanaka, T. (1979) Changes in structuredness of cytoplasmic matrix in single stimulated lymphocytes from healthy donors and patients with non-malignant and malignant diseases. *Br. J. Cancer*, **40**, 156.

Lewkonia, R. M., Kerr, E. J. L. & Irvine, W. J. (1974) Clinical evaluation of the macrophage electrophoretic mobility test for cancer. *Br. J. Cancer*, **30**, 532.

Lian, Z. J., Shi, Y. D., Chang, K. S., Chen, C. T. & Chen, W. H. (1979) An apparatus for cell electrophoresis using a square glass capillary chamber. *Acta Biochim. Biophys. Sinica* (in Chinese), **11**, 199.

Lian, Z. J. & Sea, S. B. (1980) An electronic apparatus for measuring cell electrophoretic migration time. *Acta Physiol. Sinica* (in Chinese), **32**, 92.

Light, P. A., Preece, A. W. & Ealdron, H. A. (1975) Studies with the macrophage migration inhibition (MMI) test in patients with malignant disease. *Clin. Exp. Immunol.*, **22**, 279.

Preece, A. W. & Light, P. A. (1974) The macrophage electrophoretic mobility (MEM) test for malignant diseases: Further clinical investigation and studies macrophage slowing factors. *Clin. Exp. Immunol.*, **18**, 543.

Pritchard, J. A. V., Moore, J. L., Sutherland, W. H. & Joslin, C. A. F. (1973) Technical aspects of the macrophage electrophoretic mobility (MEM) test for malignant disease. *Br. J. Cancer*, **28** (Suppl. 1), 229.

Pritchard, J. A. V., Moore, J. L., Sutherland, W. H. & Joslin, C. A. F. (1972) Macrophage-electrophoretic-mobility (MEM) test for malignant disease. *Lancet*, ii, 627.

Rawlins, G. A., Wood, J. M. F. & Bagshawe, K. D. (1976) Macrophage electrophoretic mobility (MEM) with myelin basic protein. *Br. J. Cancer*, **34**, 613.

Shaw, A., Ettin, G. & McPherson, T. A. (1976) Responses of cancer patients in MEM test: Not just a function of charge on basic protein. *Br. J. Cancer*, **34**, 713.

Shelton, J. B., Potter, C. W. & Carr, I. (1975) Cellular immunity to myelin basic protein in man and in animal model systems as measured by macrophage migration inhibition test. *Br. J. Cancer*, **31**, 528.

Shi, Y. D., Tang, Z. S., Lu, C. Z. Xiao, B. G., Lian, Z. J., Shan, Y. F., Jiang, Y. F., Yang, X. D., Lu, M. Z. & Sun, X. Z. (1979) Macrophage electrophoretic mobility test for brain tumors and malignancies. *Chinese Med. J.* (in English), **92**, 211.